# *Simplicillium lamellicola* (Smith) Zare & Gams, a novel hyperparasitic fungus infecting *Puccinia striiformis* f. sp. *tritici*

**DOI:** 10.3389/fpls.2026.1792750

**Published:** 2026-05-14

**Authors:** Mudi Sun, Xinyao Ma, Lin Yang, Yuhan Li, Huimin Tian, Zhensheng Kang, Jie Zhao

**Affiliations:** 1 State Key Laboratory of Crop Stress Resistance and High-Efficiency Production, College of Plant Protection, Northwest A&F University, Yangling, Shaanxi, China; 2 College of Plant Protection, Northwest A&F University, Yangling, Shaanxi, China

**Keywords:** bio-control, hyperparasitism, *Puccinia striiformis*, *Simplicillium*, wheat yellow rust

## Abstract

Wheat stripe (or yellow) rust, caused by the Basidiomycota fungus *Puccinia striiformis* West. f. sp. *tritici* Eriks. & Henn. (*Pst*), is one of the most destructive wheat diseases globally. Frequent outbreaks of this disease often lead to substantial crop yield losses. Currently, the primary strategies for controlling wheat stripe rust rely on the cultivation of resistant cultivars and the application of fungicides. However, fungicide use is associated with a range of adverse effects, such as environmental pollution and disruption of ecological balance. As a result, the development of promising biocontrol agents or microbial pesticides based on parasitic microbes has emerged as a more sustainable and effective approach to managing this disease. In the present study, we used a combination of morphological observation and multigenes based-molecular phylogenetic analyses to identified a hyperparasite, isolated from Pst urediospores collected from infected wheat leaves, as *Simplicillium lamellicola* (Smith) Zare & Gams. Further experimental results demonstrated that inoculating *Pst-*infected wheat leaves with *S. lamellicola* inhibited the production of *Pst* urediospores and ultimately caused the death of rust spores. These findings suggest that *S. lamellicola* holds great potential for development as a bio-control agent against wheat stripe rust, offering an eco-friendly alternative to conventional fungicides.

## Introduction

Rust diseases rank among the most destructive afflictions affecting cereal crops, including wheat (*Triticum aestivum* L.), barley (*Hordeum vulgare* L.), oat (*Avena sativa* L.), and rye (*Secale cereale* L.). Historically, these diseases have caused substantial global reduction in crop yields (Chaves et al., 2008; Zadoks, 1961). To date, rust diseases remain a major threat to cereal production (Wellings, 2011; Bouvet et al., 2022). Notably, wheat leaf rust, stripe rust, and stem rust—three primary rust diseases—have persistently impaired wheat production, leading to significant quantitative yield losses (Zadoks, 1961; Chen, 2005; Figueroa et al, 2018, Zhao and Kang, 2023). A recent study estimated that the three rust diseases cause an average annual loss of up to 15.04 million tons worldwide (Huerta-Espino et al., 2020).

Wheat stripe (or yellow) rust, caused by *Puccinia striiformis* West. f. sp. *tritici* Eriks. & Henn. (*Pst*), a fungus belonging to phylum Basidiomycota, is the most devastating of the three rust diseases affecting wheat across many regions worldwide (Wellings, 2011; Zhao and Kang, 2023). In China, since 1950, this disease has triggered eight nationwide epidemics, leading to cumulative yield losses exceeding 16.1 million tons (Liu et al., 2022). The current control strategies for wheat stripe rust, which include cultivating resistant wheat cultivars, implementing agronomic practices, and heavily relying on chemical fungicides, pose significant environmental challenges such as ecosystem contamination and potential risks to human health. Moreover, the emergence of fungicide-resistant *Pst* isolates and the disruption of natural microbial community balance have become growing additional concerns (Cook et al., 2021; Zhan et al., 2022).

Hyperparasitism is a widespread biological phenomenon among filamentous fungi. Given the significant inhibitory effects that hyperparasites exert on their host fungi, they have emerged as promising alternative to chemical fungicides to manage plant fungal diseases (Blakeman and Fokkema, 1998; Adhikari et al., 2014; Zhong et al., 2016; Zheng et al., 2017). To date, fungi from at least 30 genera have been documented to parasitize the fruiting bodies of the pathogenic rust fungi (Kapooria and Sinha, 1969; Littlefield, 1981; Yuan et al., 1999; Moricca et al., 2001; Mijušković and Vučinić, 2001; Koç and Défago, 2008; Tsuneda et al., 2011; Zhan et al., 2014; Wang et al., 2020). Among these, species from no fewer than 13 genera, including *Alternaria* Nees (Zheng et al., 2017), *Cladosporium* Link & Gray (Zhan et al., 2014; Zhang et al., 2022), *Clonostachys* Klotzsch (Wilson et al., 2020), *Epicoccum* Link (Wilson et al., 2020), *Lecanicillium* W.Gams & Zare (Wilson et al., 2020), *Microdochium* Syd (Littlefield, 1981), *Neoascohyta* Qian Chen & L. Cai (Wilson et al., 2020), *Simplicillium* Zare & W. Gams (Wang et al., 2020; Wilson et al., 2020), *Penicillium* Link (Wilson et al., 2020; Esmail et al., 2022; Wess et al., 2025), *Streptomyces* Waksman & Henrici (Zong et al., 2002), *Trichoderma* Persoon & Gray (Esmail et al., 2022), *Typhula* (Pers.) Fr (Littlefield, 1981), and *Verticillium* Nees (Mendgen, 1981), have been reported to parasitize *P. striiformis* f. sp. *tritici*. However, only one species within the *Simplicillium* genus, *S. obclavatum* (W. Gams), has been identified as a hyperparasite of *Pst* thus far (Wang et al., 2020).

*Pst* is characterized as a biotrophic parasite, entirely dependent on living host tissues for its growth and reproduction. During routine greenhouse experiments, we observed that the mycelia of a hyperparasite colonized the sporulating uredia of *Pst*-infected wheat leaves, progressively inhibiting spore production and eventually causing the complete cessation of sporulation. In the present study, we isolated and identified this hyperparasite through a combination of morphological observations (using light and scanning electron microscopy) and phylogenetic analyses based on multi-locus molecular sequencing. To confirm its parasitic activity, we conducted pathogenicity assays following Koch’s postulates and used scanning electron microscopy to observe the hyperparasite’s interaction with *Pst* urediospores on infected wheat leaves. These findings lay a foundation for further evaluation of the hyperparasite’s potential as a fungal biocontrol agent against managing wheat stripe rust.

## Materials and methods

### Plant and fungal materials

Wheat cultivar Mingxian 169, which is highly susceptible to all known races of *Pst* and routinely used for *Pst* recovery and propagation, was grown in pots containing a commercial potting mix in a rust-free, climate-controlled growth chamber within a greenhouse facility. The growth chamber was equipped with a dual-regulation system enabling the precise control of temperature and light conditions. Then, 10-day old seedlings were inoculated with *Pst* race CYR34. Following the inoculation, these plants were used for *in vivo* assays to evaluate the hyperparasite parasitism of *Pst* as well as for subsequent microscopic observations.

### Isolation and purification

To isolate the hyperparasite, three leaf samples were collected from *Pst-*infected wheat plants showing hyperparasite colonization; these plants were grown in separate pots in the climate-controlled greenhouse growth chamber. Each leaf sample was cut into two 1-cm segment at the boundary of the *Pst* uredia: one segment with visible white hyperparasite mycelium and one without. The leaf segments were surface-sterilized by immersion in 75% (v/v) ethanol solution, rinsed three times with sterile water, and then placed onto potato dextrose agar (PDA) plates using autoclaved forceps in a laminar flow hood. As a control, healthy wheat leaf segments were processed with the same sterilization and plating protocol. All plates were incubated at 25°C for 6 days to allow colony formation. Pure cultures of the hyperparasite were obtained by transferring mycelial plugs from the edge of emerging colonies onto fresh PDA plates, followed by incubation under the same conditions as stated above.

### Experimental design

To determine whether the hyperparasite is able to colonize *Pst* urediospores, the pure culture of the hyperparasite was inoculated with urediospore-producing wheat leaves as per the following procedure: Approximately 1 mg of urediospores of the *Pst* race CYR34 was suspended in 1 mL of methoxy-perfluorobutane fluid (Novec™ 7100, 3M, USA) in a 2-mL microcentrifuge tube to prepare a spore suspension. After thorough mixing, 10-day old wheat seedlings were inoculated with the spore suspension following a standard method and incubated under the same conditions as described previously (Ju et al., 2022). At 11 days post-inoculation (dpi), when the *Pst-*infected wheat leaves were actively sporulating, they were used for hyperparasite inoculation. A spore suspension of the hyperparasite isolate YL23 was prepared by scraping the surface of a PDA culture into a 5-mL microcentrifuge tube containing stirred water and gently stirring with a sterile glass rod in a laminar flow hood. The mixture was centrifuged at low speed for 30 s at room temperature, and the supernatant was transferred into a 5-mL atomizer under aseptic conditions. The resulting suspension (1.2 × 10^5^ spores/mL) was sprayed onto the surface of *Pst*-sporulating wheat seedlings. The inoculated plants were covered with a clean transparent plastic cylinder, and the top was sealed with a lid after spraying sterile water inside to maintain the humidity. They were then placed in a dew chamber (Percival Scientific, Inc., IA, USA) and incubated at 16°C in the dark with high relative humidity (>80% RH) for 24 h. After incubation, the plants were transferred to a clean growth chamber under a controlled photoperiod and temperature regime: 16°C for 16 h (day) and 13°C for 8 h (night). The wheat seedlings inoculated with *Pst* race CYR 34 only were maintained under the same conditions as a negative control. Three experimental repetitions were performed. Once visible white mycelia were observed to be growing on the urediospore surface, the white mycelia were isolated following the aforementioned method.

### Morphological observation

To observe morphological characteristics, mycelial fragments were collected from the edge of a freshly cultured pure hyperparasite colony. These fragments were placed in a drop of sterile water on a clean glass slide and gently dispersed using a sterile inoculation needle. A coverslip was then placed over the preparation, and the slide was mounted on the stage of a light microscope (BX51T-32P01, Olympus, Japan). Using this setup, we examined the morphological features of the hyperparasite’s hyphae and conidia and measured the dimensions of 50 conidia to obtain size statistics.

After inoculation with *Pst*, white mycelial growth was observed on the uredia of infected wheat leaves, and these leaves were collected as experimental specimens. Each specimen was cut into 0.5 × 0.5-cm^2^ squares, placed in 2-mL microcentrifuge tubes containing 4% (v/v) glutaraldehyde fixative, and incubated at 4°C for 16 h for fixation. Subsequently, all specimens were rinsed four times with 0.1 M PBS buffer (pH 6.8), with each rinse lasting 10 min and performed in a fume hood. The fixed specimens were then dehydrated through a graded ethanol series (30%, 50%, 70%, 80%, and 90%, v/v), with each concentration step maintained for 15 to 30 min in the fume hood. Finally, the specimens were treated three times, with the treatment lasting 30 min. Following dehydration, all specimens were immersed in isoamyl acetate solution (v/v) for 10 to 20 min as per the method described by Bomblies et al. (2008). Morphological observations of the hyperparasite–*Pst* interaction were conducted using a Hitachi S-4800 scanning electron microscope (SEM, Japan).

### DNA extraction, PCR amplification, and sequencing

Pure cultures of the hyperparasite were transferred onto fresh PDA plates in a laminar flow hood, sealed with parafilm, and incubated at 25°C until colonies covered nearly the entire plate surface. Mycelia were then harvested by scraping with a sterile scalpel in the hood. The collected mycelia were frozen at -20°C and subsequently lyophilized using a vacuum freeze dryer. Genomic DNA was extracted from the lyophilized mycelia using a fungal genomic DNA extraction kit (Beijing Solarbio Sci-Tech. Co., Ltd., China). To identify the hyperparasite, the internal transcribed spacer (ITS) region was amplified using universal primers ITS1 (5′-TCCGTAGGTGAACCTGCGG-3′) and ITS4 (5′-TCCTCCGCTTATTGATATGC-3′) (White et al., 1990). The 25-μL PCR reaction contained 1 μL of each ITS primer (10 mM), 2 μL of genomic DNA template (50 ng/μL), 12.5 μL of 2×PCR Master Mix (Shanghai Beyotime Bio-Tech., Co., Ltd., China), and 8.5 μL of sterile deionized water. To enhance the accuracy of species identification, two additional conserved gene regions were amplified: elongation factor-1 alpha (*TEF1-α*) using primers 983F (5′-GCYCCYGGHCAYCGTGAYTTYAT-3′) and 2218R (5′-ATGACACCRACRGCRACRGTYTG-3′) (Rehner and Buckley, 2005) and RNA polymerase II subunit B 1 (RPB1) using primers CRPBaf (5′-GARTGYCCDGGDCAYTTYGG-3′) and RPB1Cr (5′-CCNGCDATNTCRTTRTCCATRTA-3′) (Castlebury et al., 2004; Medeiros et al., 2021). All PCR amplifications were performed using the following thermal cycling program: initial denaturation at 94°C for 5 min, 40 cycles of denaturation at 94°C for 30 s, annealing at 60°C for 40 s, and extension at 72°C for 40 s, followed by a final extension at 72°C for 7 min and a final hold at 10°C until retrieval. Sterile deionized water was included as a negative control in each PCR run. The resulting PCR amplicons were separated by being electrophoresed on 1% (m/v) agarose gel at 90 V for 40 min. The target bands were excised, purified using a PCR purification kit (Shanghai Beyotime Bio-Tech., Co., Ltd., China), and sequenced by using Sangon Biotech (Shanghai) Co., Ltd. (Shanghai, China).

### Phylogenetic analysis

To confirm the taxonomic identity of the hyperparasite isolate YL23, we performed a sequence alignment of its assembled genome using BLAST against the GenBank database available in the NCBI database (https://www.ncbi.nlm.nih.gov/). For a detailed phylogenetic placement, we also downloaded representative sequences of *Simplicillium* species and other related genera from NCBI. These sequences were aligned using the default parameters of the multiple sequence alignment tool in MEGA 11 software (https://megasoftware.net). Subsequently, sequence clustering was conducted under the maximum-likelihood (MLE) method implemented in MEGA 11. A phylogenetic tree was constructed with 1,000 bootstrap replications to assess the robustness of the inferred clades, following standard methodologies (Tamura et al., 2021).

### Hyperparasitism experiment

To confirm the pathogenicity of the hyperparasite, isolate YL23 was used to inoculate *Pst*-sporulating wheat leaves using the aforementioned inoculation method. The same incubation and cultivation conditions were employed. Leaf segments were collected at five time points, 24, 48, 72, 96, and 120 h after the hyperparasite isolate YL23 inoculation, and examined using SEM. All leaf samples were prepared for SEM observation using the method described earlier in this study.

## Results

### Isolation of the hyperparasite

**Figure 1:**
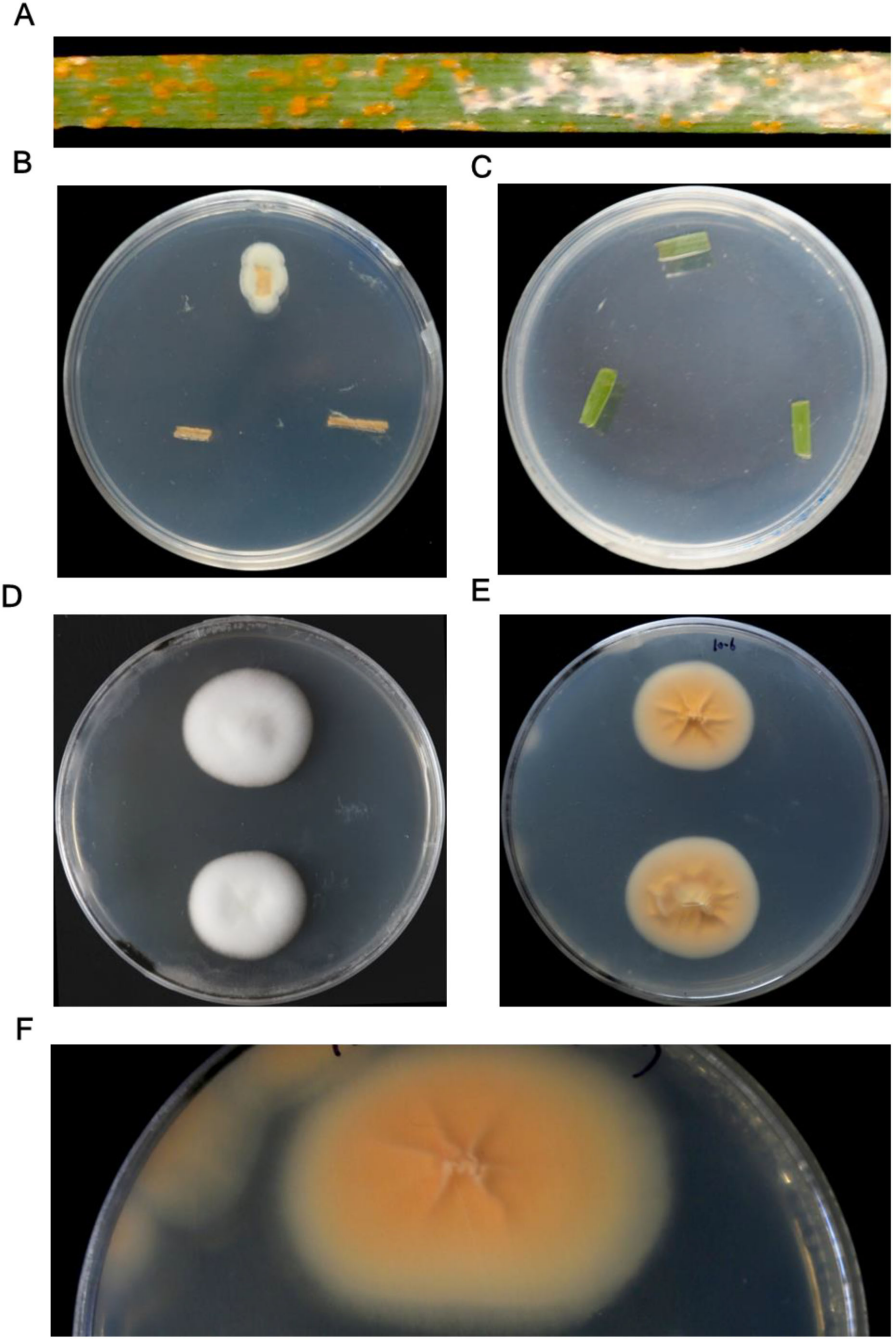
Images showing the isolation and colony features of the hyperparasite *Simplicillium lamellicola* on potato dextrose agar (PDA) medium. **(A)** White mycelial growth of the hyperparasite *S. lamellicola* colonizing a wheat leaf infected with *Puccinia striiformis* f. sp. *tritici* (*Pst*). **(B)** Colony of *S. lamellicola* growing on PDA after 6 days of incubation at 25°C. **(C)** Healthy wheat leaf segment (control). **(D, E)** Front (obverse) and reverse views of *S. lamellicola* colonies on PDA medium after 15 days at 25°C, respectively. **(F)** Close-up view of a single colony.

The hyperparasite initially appeared as white, loose mycelia on the uredia of *Pst*-infected leaves, which subsequently became denser over time (**Figure 1A**). As the mycelia aged, their color shifted from white to gray. The colonization ultimately led to the cessation of *Pst* sporulation and the inactivation of *Pst* urediospores. Initially thought to be an occasional occurrence, this phenomenon was later observed repeatedly. To identify the hyperparasite, four isolates with uniform colony morphology were recovered from the collected leaf samples on PDA plates after 6 days of incubation at 25°C. In contrast, no colonies were obtained from healthy leaf segments (**Figures 1B, C**). On PDA, the hyperparasite isolate YL23 formed white, flocculent colonies over 15 days at 25°C, exhibiting a villiform (velvety) appearance when viewed from above (**Figure 1D**). The reverse side of the colonies appeared pale yellow with distinct radial ridges extending from the center to the edge. As the colony growth progressed, the reverse color deepened to orange (**Figure 1E**). The hyperparasite grew slowly on PDA, and mature conidia were readily released, often giving rise to new satellite colonies on the same plate (**Figure 1F**).

After being artificially inoculated onto the urediospore surface of *Pst*-infected wheat leaves, the pure culture of the hyperparasite successfully colonized the *Pst* urediospores and produced white mycelia. These white mycelia were subsequently isolated, and their colony morphology was consistent with that of the aforementioned isolates.

### Taxonomic identification of the hyperparasite

**Figure 2:**
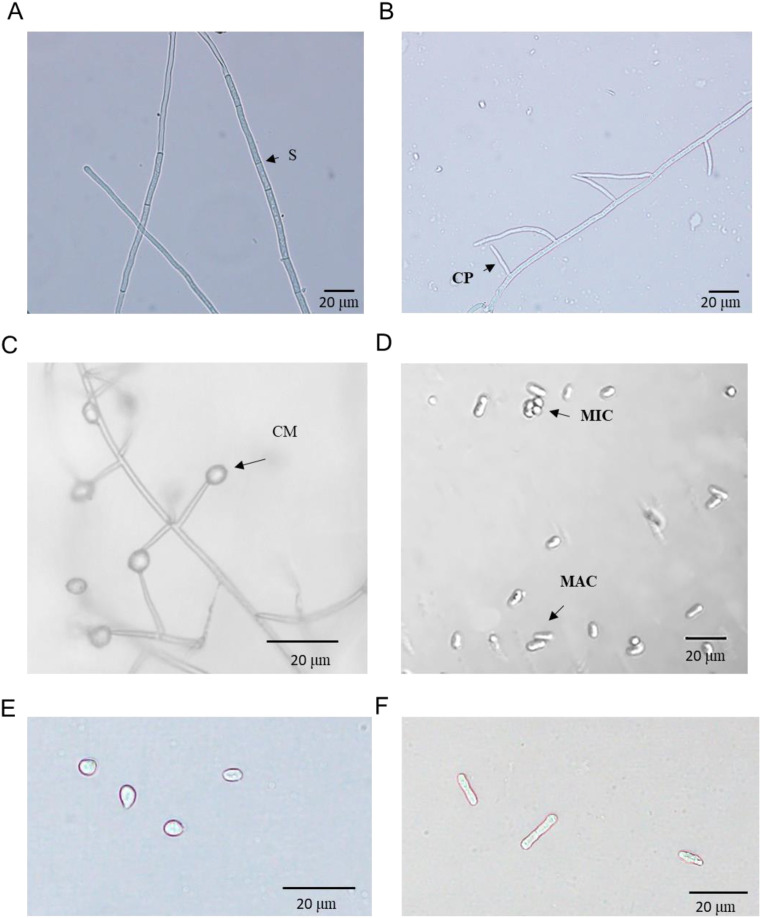
Observations of *Simplicillium lamellicola* under light microscopy. **(A)** Septate (S) hyphae. **(B)** Conidiophores (CP). **(C)** Conidial masses (CM) at the apex of conidiophores. **(D)** Macroconidia (MAC) and microconidia (MIC). **(E, F)** Close-up views of microconidia and macroconidia, respectively.

**Figure 3:**
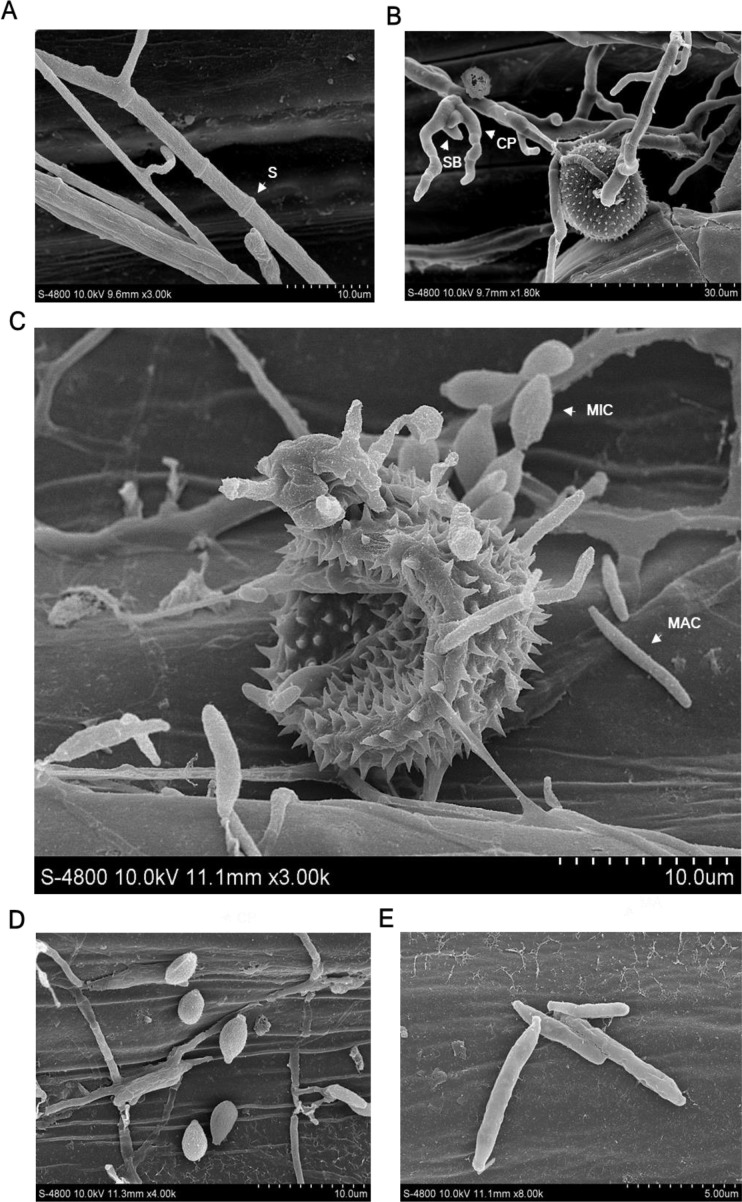
Observations of *Simplicillium lamellicola* under light and scanning electron microscopy. **(A)** Hyphae with septa (S). **(B)** Conidiophores (CP) with secondary branches (SB). **(C)** Microconidia (MIC) and macroconidia (MAC). **(D, E)** Close-up views of microconidia and macroconidia, respectively.

The isolate YL23 was identified through morphological observation and phylogenetic analysis. Light and scanning electric microscopy revealed septate hyphae (S) (**Figures 2A**, **3A**). Conidiophores (CP, also phialides) arose from the hyphae and tapered toward the apex. They were hyaline and aseptate, measuring 10 to 32 μm in length (average, 21 μm). Most conidiophores were solitary, though some occurred in pairs or even in verticillate clusters (**Figures 2B, C**, **3B**). In some cases, the conidiophores produced secondary branches (SB) (**Figure 3B**). Spherical conidial masses (CM, heads) formed at the apex of the conidiophores (**Figure 2C**). Based on the measurement of 30 spores, the microconidia (MIC) were oval and hyaline with dimensions of 2.7–4.8 μm × 3.3–1.1 μm (average, 3.9×1.6 μm) (**Figures 2D, E**, **3C, D**, **Supplementary Table 1**) and the macroconidia (MAC) were oblong, hyaline, typically straight, and measured 7.2–14.9 μm × 0.8–1.8 μm (average, 10.6 μm×1.4 μm) (**Figures 2D, F**, **3C, E**, **Supplementary Table 1**). These morphological characteristics align with the description of *Simplicillium lamellicola* (Smith) Zare & Gams provided by Zare and Gams (2001), supporting the taxonomic assignment of the isolate YL23 to the genus *Simplicillium*. To our knowledge, this species has not been previously reported as a hyperparasite of *Pst*.

**Figure 4:**
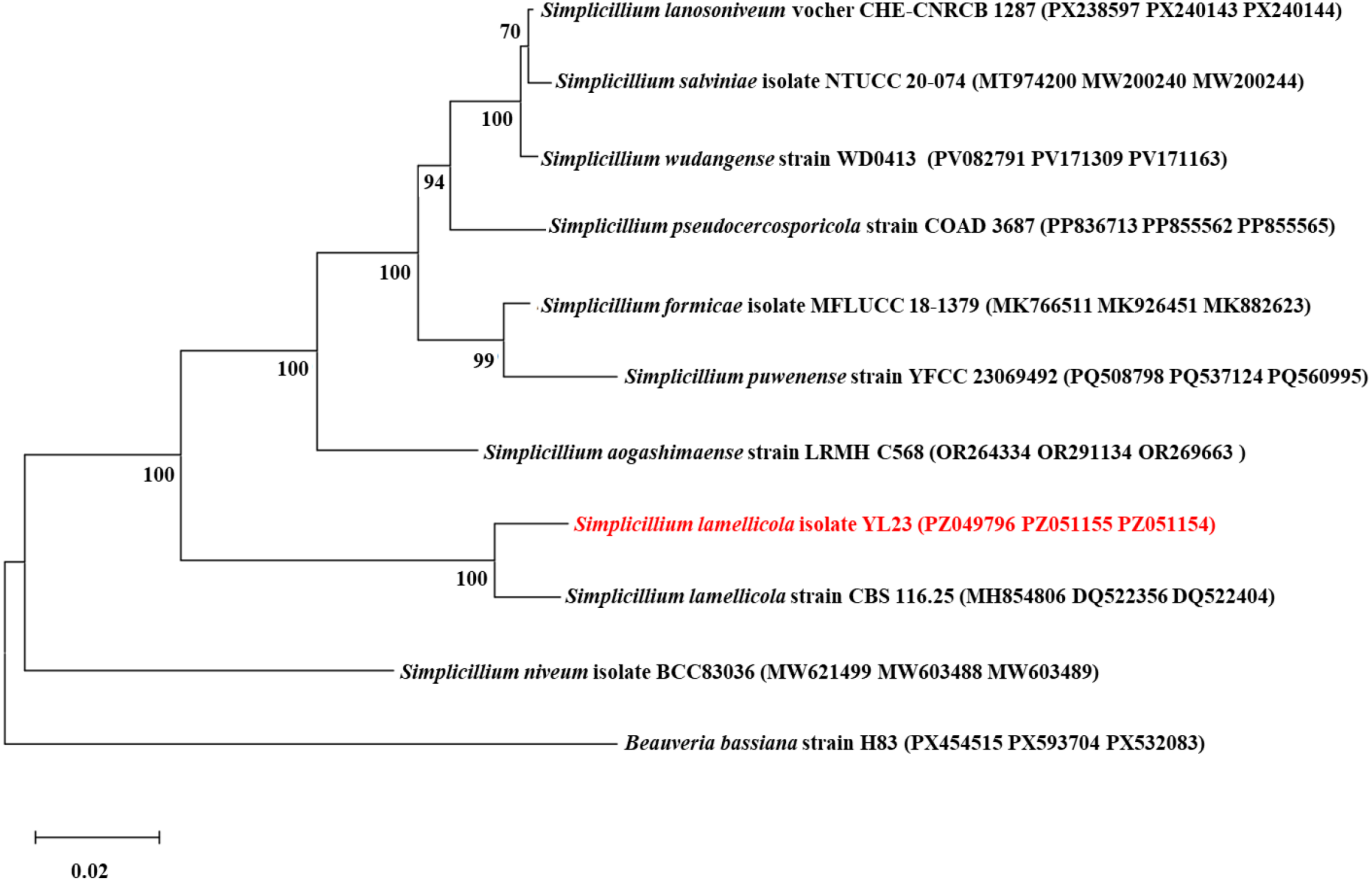
A maximum-likelihood phylogenetic tree was constructed from concatenated ITS, *TEF1-α*, and RPB1 sequences using MEGA 11. The tree depicts the genetic placement of the *Simplicillium lamellicola* isolate YL23 relative to other species in the genera of *Simplicillium* and *Beauveria* (used as the outgroup). Bootstrap support values based on 1,000 replications are indicated at the nodes.

The DNA sequences of the ITS region, *TEF1-a*, and RPB1 were obtained from the isolate YL23 through PCR amplification. These sequences have been deposited in the GenBank database under the following accession numbers: PZ049796 (ITS), PZ051155 (*TEF1-a*), and PZ051154 (RPB1). Phylogenetic analysis based on the concatenated ITS/*TEF1-a*/RPB1 sequences placed the isolate YL23 in a clade closely related to *S. lamellicola* strain CBS 116.25 (GenBank accession numbers MH854806, DQ522356, and DQ522404). However, it formed distinct clades separate from other ingroup *Simplicillium* species and the outgroup species *Beauveria bassiana* [Bals.-Criv.) Vuill.] (**Figure 4**). Combined with morphological characteristics, these results confirm that the isolate YL23 belongs to the species *Simplicillium lamellicola* (Smith) Zare & Gams within the phylum Ascomycota.

### *In vivo* test of hyperparasitism of *S. lamellicola* infecting *Pst*

**Figure 5:**
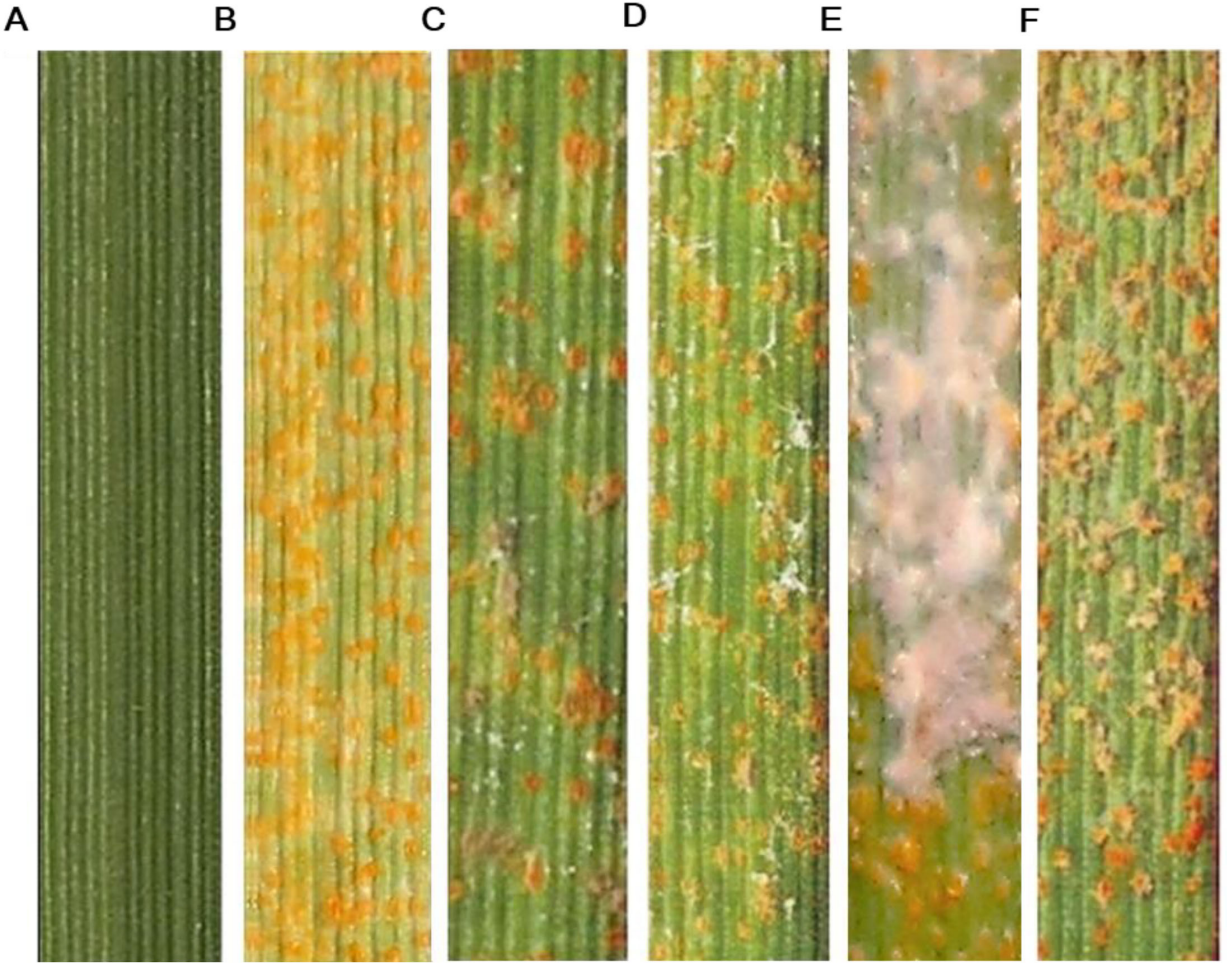
Images showing the pathogenicity of *Simplicillium lamellicola* toward *Puccinia striiformis* f. sp. *tritici* (*Pst*) on wheat leaves (cv. Mingxian 169). **(A)** Asymptomatic wheat leaves at 14 days post-inoculation (dpi) with the *S. lamellicola* isolate YL23. **(B)** Wheat leaves showing the yellow uredia of *Pst* at 14 dpi with the race CYR34. **(C**–**F)** Symptoms of *Pst* uredia and *S. lamellicola* mycelia observed at 3, 5, 7, and 11 dpi, respectively, following the inoculation of wheat leaves (pre-infected with *Pst* for 12 days) with the *S. lamellicola* isolate YL23.

To evaluate the ability of the *S. lamellicola* isolate YL23 to parasitize *Pst*, a hyperparasitism assay was conducted. Wheat leaves (cv. Mingxian 169) inoculated with a spore suspension of the isolate YL23 remained asymptomatic 20 days post-inoculation (dpi) (**Figure 5A**). In contrast, leaves inoculated only with the *Pst* race CYR34 displayed typical uredia symptoms (**Figure 5B**). In a separate treatment, wheat leaves were first inoculated with the *Pst* race CYR34, followed 12 days later by inoculation with a spore suspension of the *S. lamellicola* isolate YL23. At 3 dpi with the hyperparasite, abundant white mycelia growth was clearly observed on *Pst* uredia. The mycelia coverage increased noticeably by 5 and 7 dpi, indicating the rapid proliferation of the hyperparasite (**Figures 5C–E**). By 11 dpi, the mycelia had changed from white to gray and began to decline in density. Concurrently, *Pst* sporulation on wheat leaves decreased sharply and eventually ceased (**Figure 5F**). These results suggest that *S. lamellicola* isolate YL23 is a novel hyperparasite of *Pst* and possesses potential as a fungal biocontrol agent to manage wheat stripe rust.

### Microscopic observations of *S. lamellicola* parasitizing *Pst*

**Figure 6:**
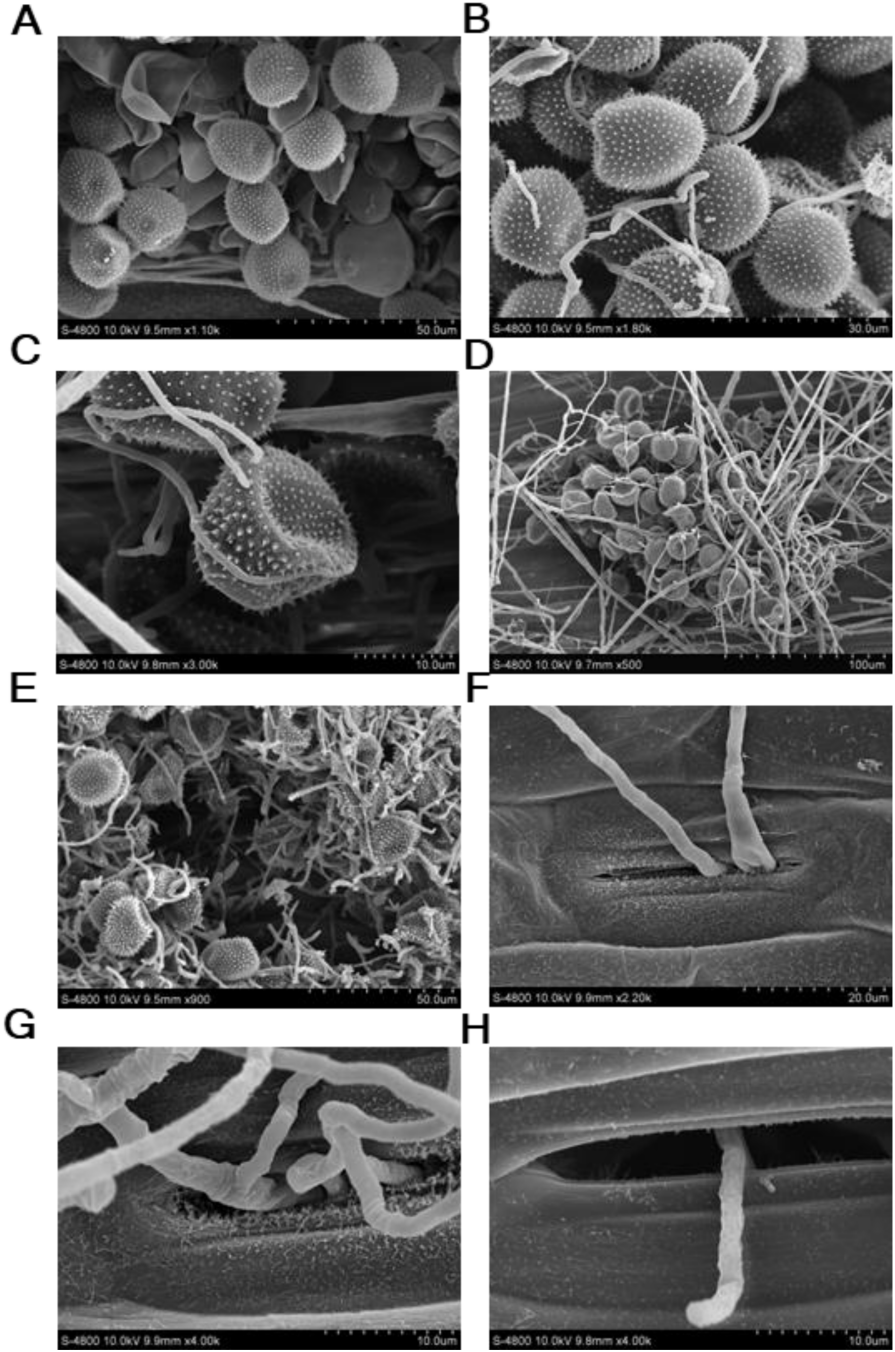
Scanning electron microscope observations of *Simplicillium lamellicola* parasitizing *Puccinia striiformis* f. sp. *tritici* (*Pst*) on the leaves of wheat cultivar Mingxian 169. **(A)** Uredia of *Pst* without *S. lamellicola* infection. **(B)** Hyphae of *S. lamellicola* growing among urediospores within *Pst* uredia. **(C)** Close-up view of *S. lamellicola* colonizing the surface of *Pst* urediospores. **(D)** Hyphae of *S. lamellicola* covering *Pst* uredia on the surface of wheat leaves. **(E)** Hyphae crossing the inter-spore spaces within a *Pst* uredium. **(F, G)** Hyphae of *S. lamellicola* penetrating wheat leaf tissues through a stoma. **(H)** Hyphae of *S. lamellicola* emerging from a stomal slit on the wheat leaf surface.

To further confirm the hyperparasitic activity of the *S. lamellicola* isolate YL23 parasitizing *Pst*, SEM was performed. Observations revealed that, following co-inoculation with the *Pst* race CYR34 and the isolate YL23, the hyphae of the *S. lamellicola* isolate YL23 rapidly proliferated among urediospores within *Pst* uredia, in contrast to the YL23-uninoculated control leaves infected solely with the *Pst* race CYR34 (**Figures 6A, B**). Upon contact with *Pst* urediospores, the hyphae of the isolate YL23 rapidly colonized their surfaces (**Figure 6C**). As the hyperparasite developed, the hyphal network became denser, gradually covering the *Pst* uredia and permeating the inter-spore spaces (**Figures 6D, E**). Interestingly, the hyphae of the isolate YL23 were not only capable of penetrating wheat leaf tissues through the stomata (**Figures 6F, G**) but also observed emerging from stomatal openings (**Figure 6H**).

**Figure 7:**
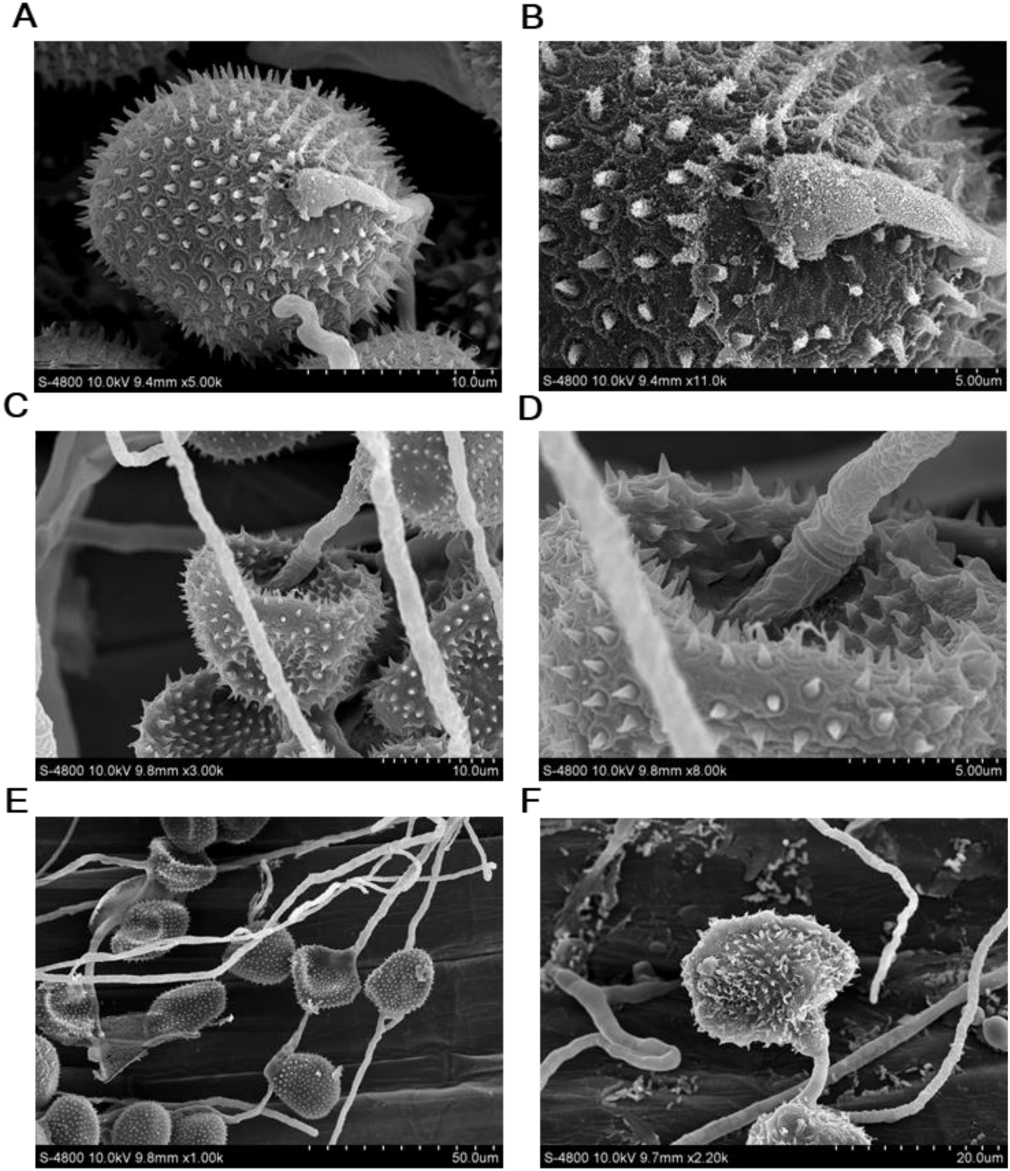
Scanning electron microscope observations of the penetration of *Simplicillium lamellicola* into the urediospores of the *Puccinia striiformis* f. sp. *tritici* (*Pst*) race CYR34 on the surface of wheat leaves (cv. Mingxian 169). **(A, B)** Inflated top of the hyphae of *S. lamellicola* on the surface of a urediospore of *Pst* and a close-up view prior to penetration into the urediospores. **(C, D)** A urediospore of *Pst* showing the appearance of obvious invagination after penetration by a hypha of *S. lamellicola* and a close-up view. **(E)** Hyphae of *S. lamellicola* penetrated the urediospores of *Pst*, resulting in partial or almost complete invagination. **(F)** A dying urediospore caused by the penetration of hyphae of *S. lamellicola* and disaffiliated thorns from the surface of the urediospore.

When the hyphal tips of the isolate YL23 reached the germ pores on the surface of urediospores of the *Pst* race CYR34, they swelled to form an appressorium-like infection structure and penetrated into the urediospores (**Figures 7A, B**). The hyphae then absorbed contents from the inside of the urediospores, causing visible invagination of the urediospores by 3 dpi (**Figures 7C, D**). By 5 and 7 dpi, the hyphae of the *S. lamellicola* isolate YL23 had extensively penetrated the urediospores, absorbing their contents to the extent that the spores became flattened and pancake-like in appearance (**Figure 7E**). This process led to the collapse of the urediospores and the detachment of the surface spines (**Figure 7F**). These results demonstrated that the *S. lamellicola* isolate YL23 exhibits a clear hyperparasitic ability against *Pst*.

## Discussion

Nearly a century ago, *S. lamellicola* was originally described as *Cephalosporium lamellicola* F.E.V. Sm., an anamorphic fungus found parasitizing the edible mushroom *Agaricus bisporus* (J.E. Lange) Imbach in England (Smith, 1924).The species was later redefined and transferred to *Verticillium lamellicola* (F.E.V. Sm.) W. Gams in 1971. It is currently recognized under the name *Simplicillium lamellicola* (F.E.V. Sm.) Zare & W. Gams, following the reclassification by Zare and Gams (2001) into the newly established genus *Simplicillium* Zare & Gams (https://www.indexfungorum.org). To date, this species has been documented as a parasitize of various organisms, including rust fungi, polypores, insects, and nematodes (Zare and Gams, 2001; Gams and Zare, 2003). It also exhibits a broad geographical distribution worldwide. In the present study, we demonstrate for the first time that *Simplicillium lamellicola* acts as a hyperparasite on *Pst*. This hyperparasitic interaction leads to the disruption of sporulation and the death of *Pst* urediospores following colonization. However, there is no reported evidence to date that this species can infect other cereal rust species, such as *P. striiformis*, *P. triticina*, and *P. graminis*.

*S. lamellicola* also exhibits robust hyperparasitic activity against a broad range of phytopathogenic fungi, such as *Leptosphaeria biglobosa* Shoemaker & H. Brun (the causal agent of blackleg of oilseed rape), *Botrytis cinerea* Pers. (gray mold), *Sclerotinia sclerotiorum* (Lib), de Bary (stem rot) (Shin et al., 2017; Li et al., 2023), *Thielaviopsis paradoxa* (Berk. & M.A. Curtis) Sacc. (stem decay), and *Heterobasidion annosum* (Fr.) Bref. (root and stem decay of conifers) (Azeez et al., 2026). This hyperparasitic capacity is attributed to the fungus’ ability to produce multiple antifungal metabolites, such as aureobasidin A1 and squalestatin S1, during the infection process (Li et al., 2023). In addition to its hyperparasitic properties, *S. lamellicola* has demonstrated significant efficacy in controlling various plant diseases. Shin et al. (2017) reported that the *S. lamellicola* isolate BCP effectively suppressed gray mold on tomato and ginseng. The isolate 16047 of this species was validated to inhibit *Fusarium graminearum* Schwabe, the pathogen responsible for fusarium head blight, reducing disease severity across multiple experimental conditions while also promoting wheat growth (Abaya et al., 2021). This isolate has since been developed into a microbial biopesticide that strongly inhibits the growth of diverse fungal and bacterial plant pathogens (Dang et al., 2014). Recent research has focused on the transcriptomic and genomic sequencing of *S. lamellicola* and *S. aogashimaense* Y. Takahashi & K. Takamatsu (Li et al., 2023; Zhu et al., 2022; Azeez et al., 2026). These results not only deepen our understanding of the fungus-to-fungus interaction mechanism underlying hyperparasitism but also facilitate the development of more targeted microbial biopesticides. Notably, several known hyperparasites of *Pst* have been identified, including 13 genera mentioned in the introduction of this study, *S. obclavatum* (W. Gams) Zare & W. Gams (Wang et al., 2020), and now *S. lamellicola* as reported here. These species hold great potential as candidates for the development of microbial biopesticides or biocontrol agents against wheat stripe rust, though further research is needed to fully explore their applications.

Hyperparasitic species within the genus *Simplicillium* exhibit a broad host range of organisms, with documented roles not only as hyperparasites of rust fungi but also as entomopathogens. Notably, *S. lanosoniveum* (J.F.H. Beyma) Zare & W. Gams has been identified as a natural hyperparasite of *Phakopsora pachyrhizi* Syd. & P. Syd. (the causal agent of soybean rust) in the field (Ward et al., 2011). The *S. lanosoniveum* isolate IS698–2 is capable of parasitizing *P. graminis* Pers. f. sp. *tritici* Eriks. & Henn., the pathogen responsible for wheat stem rust, and can significantly impair urediospore sporulation and germination in this rust species (Si et al., 2022). However, there are no existing records of *S. lanosoniveum* parasitizing *Pst*. Most recently, the *S. lanosoniveum* isolate LBSIM-01, collected from coffee leaf rust lesions in Cuba, was formally reported for the first time (Robaina et al., 2024). The *S. aogashimaense* isolate HWYR21 has been validated both *in vitro* and *in vivo* to effectively reduce uredial formation and strongly inhibit urediospore germination across multiple rust species, including *P. triticina* Erikss., *P. hordei* G.H. Otth., and *P. coronata* Corda f. sp. *avenae* Erikss (Wilson et al., 2020). Subsequently, *S. aogashimaense* was also documented as a hyperparasite of *Blumeria graminis* (DC.) Speer f. sp. *tritici*, the causal agent of wheat powdery mildew (Zhu et al., 2022). As for *S. obclavatum*, the isolate RV26, recovered from *Pst* uredia, has been shown to induce urediospore mortality by disrupting fruiting body sporulation in this rut pathogen (Wang et al., 2020). In this study, we verified the hyperparasitic ability of *S. camellicola* under laboratory conditions. However, its field application requires a further study in the future, as environmental factors such as high humidity may impose significant limitations. Therefore, identifying the key genes or substance underlying the pathogenic process triggered by this fungus and developing them into stable biocontrol agents could represent a practical and feasible strategy.

*Simplicillium* species are capable of parasitizing the aecia formed on the tissues of alternate (aecial) hosts of certain rust fungi. In a previous report, *S. lanosoniveum* was found to be naturally occurring on *Aecidium elaeagni-latifoliae* Petch in India and confirmed to act as a hyperparasite of this rust stage (Baiswar et al., 2014). Whether *S. camellicola* can parasitize *Pst* at the aecial stage on its alternate hosts requires further investigation.

## Conclusions

To sum up, the present study is the first to report that *Simplicillium lamellicola* acts as a hyperparasite of *Puccinia striiformis* f. sp. *tritici* (*Pst*) based on morphological observations with light and scanning electron microscopy combined with molecular and biological analyses. This finding adds *S. lamellicola* to the known roster of hyperparasite targeting *Pst*. Morphologically, *S. lamellicola* produces macroconidia and microconidia borne at the apex of verticillate conidiophores. During parasitism, it penetrates *Pst* urediospores through their germ pores, extracting cellular contents and causing severe invagination of the spore structure, which can lead to the complete destruction of the urediospores. Additionally, the hyperparasite is capable of entering wheat leaf tissues via the stomata and emerging through the same route to spread. *In vivo* assays further confirmed the robust hyperparasitic activity of *S. lamellicola* against *Pst*. Given these characteristics, *S. lamellicola* holds significant potential for development as biocontrol agents or microbial pesticide not only to manage wheat stripe rust but also to control other rust diseases affecting a broad range of plant species.

## Data Availability

The data supporting the conclusion in the present work are included in the article/supplementary material. Further inquiries can be directed to the corresponding author.
