# Supplementary material for: Simplicillium lamellicola (Smith) Zare & Gams, a novel hyperparasitic fungus infecting Puccinia striiformis f. sp. tritici
Source: Front Plant Sci. 2026 May 14;17:1792750. doi: 10.3389/fpls.2026.1792750 (PMC13217696; doi:10.3389/fpls.2026.1792750)
Supplement: Supplementary file 1 [file Table1.pdf]

Supplementary Table 1 The size of macrospores and microspcore of *Simplicillium lamellicola*(Smith) Zare & Gams isolate YL23 measured under a light microscope

No.	Macrospores		No.	Microspores	
	Length (μm)	Width (μm)		Length (μm)	Width (μm)
1	12.17	1.25	1	3.67	1.78
2	8.92	1.49	2	3.84	1.32
3	9.21	1.15	3	4.81	1.28
4	7.16	0.83	4	3.5	1.33
5	12.60	1.43	5	4.27	1.13
6	13.43	1.23	6	4.5	2.65
7	8.55	1.28	7	4.12	1.50
8	9.92	1.35	8	3.7	1.70
9	14.10	1.7	9	3.68	1.54
10	8.14	1.31	10	3.45	1.48
11	14.79	1.66	11	4.32	2.01
12	8.15	1.26	12	3.54	1.58
13	13.48	1.55	13	3.66	1.54
14	11.55	1.17	14	4.21	1.65
15	8.29	1.42	15	4.37	1.68
16	9.81	1.35	16	3.36	1.53
17	8.08	1.03	17	4.34	1.78
18	11.73	1.16	18	3.86	1.51
19	8.05	1.25	19	3.32	1.32
20	8.92	1.33	20	3.71	1.33
21	8.24	1.64	21	4.25	2.35
22	7.46	1.56	22	4.1	1.66
23	9.17	1.78	23	3.84	1.46
24	12.58	1.59	24	3.56	1.78
25	13.15	1.78	25	3.28	1.59
26	10.78	1.30	26	4.15	1.74
27	14.92	1.15	27	3.69	1.42
28	10.65	1.75	28	4.08	1.86
29	12.46	1.27	29	3.77	1.84
30	11.55	1.12	30	3.51	1.65
Averge	10.6	1.4	Averge	3.9	1.6
Maximum	14.9	1.8		4.80	2.7
Minimum	7.2	0.8		3.30	1.1
